# Antibiotic resistance and ESBL production in *Escherichia coli* from various sources in Aba metropolis, Nigeria

**DOI:** 10.1186/s42269-021-00628-5

**Published:** 2021-10-19

**Authors:** Martha Uzoaru Ajuga, Kome Otokunefor, Obakpororo Ejiro Agbagwa

**Affiliations:** 1 Department of Microbiology, Abia State Polytechnic, P.M.B 7166, Aba, Nigeria; 2 Department of Microbiology, University of Port Harcourt, P.M.B.5323, Port Harcourt, Nigeria

**Keywords:** Multidrug resistance, ESBL, *Escherichia coli*, Nigeria

## Abstract

**Background:**

The increase in multidrug resistance (MDR) among pathogenic bacteria responsible for infectious diseases has led to lack of effectiveness of some antibiotics. The ability of *Escherichia coli* to harbor resistant genes has made the treatment of infections a major challenge. This study was carried out to assess antibiotic resistance and extended-spectrum beta-lactamase (ESBL) production of E. coli from various sources in Aba metropolis, Nigeria.

**Results:**

From a total of 350 samples collected from clinical and non-clinical sources, 137 were presumptively identified as *E. coli* by standard phenotypic methods and 83 were confirmed as *E. coli* by the detection of *E. coli* specific 16S rRNA gene fragments. The majority of these isolates (52, 62.7%) were from non-clinical sources. The clinical isolates, however, exhibited a higher level of resistance against 62.5% of tested antibiotics. Both group of isolates exhibited similar levels (58.1% vs 53.9%) of MDR, though. A low rate of ESBL production was observed (1.2%) following phenotypic detection of ESBL-producing abilities using the double-disc synergy test. An assessment of the presence of three beta-lactamase gene genotypes (bla_TEM_, bla_SHV_ and bla_CTX-M_) revealed that none of the three predominant ESBL genotypes was identified in this study.

**Conclusions:**

This study reports high levels of antibiotic resistance in both clinical and non-clinical *E. coli* isolates. Though higher rates of resistance were observed among the non-clinical isolates, both group of organisms had similar levels of MDR. Strikingly, however, was the low level of ESBL producers detected in this study and the absence of the three main genotypes associated with ESBL production in this study.

## Background

Extended-spectrum beta-lactamase (ESBL)-producing bacteria are one of the more recent bacterial evolutions observed in the ongoing antibiotic-resistant pandemic. Since the first detection of these organisms, an increasing prevalence of ESBL-producing bacteria has been described worldwide (Denis et al. 2015). These particular groups of resistant bacteria are important because the extended-spectrum beta-lactamase enzymes confer on the producing organism resistance to a wider spectrum of beta-lactam antibiotics, key of which are the third-generation cephalosporins. These were at the forefront in treating beta-lactamase drug-resistant bacteria (Paterson and Bonomo 2005). ESBL production has also been associated with increasing levels of drug failure, longer hospital stays, cost, prognosis and mortality in some cases (Nivesvivat et al. 2018; Sakellariou et al. 2016; Wilson and Torok 2018; Mita et al. 2019).

*Escherichia coli* and *Klebsiella pneumoniae* have been described as the two predominant groups of bacteria associated with ESBL production. Initial reports of ESBL-producing bacteria were associated with nosocomial outbreaks, but over time spread to the community (Wilson and Torok 2018). The ESBLs themselves are generally of different types. The less commonly reported ESBLs include the OXA type, PER type and GES type (Shaikh et al. 2015). Those belonging to the SHV, TEM and CTX-M families have, however, been described as the most commonly encountered among clinical isolates (Bradford 2001; Mahamat et al. 2019; Malande et al. 2019). A recent review study (Tanko et al. 2020) on ESBL-producing Gram-negative bacteria in Nigeria similarly reports on the detection of these predominant ESBL genes. The study carried out between January 2004 and November 2019 retrieved 217 articles published within that time period, but only 60 reported on both the phenotypic and genotypic detection of ESBL production in human isolates. More research related to this is therefore essential. Studies exploring this phenomenon will result in more robust local epidemiological data which is key in a proper characterization of contribution of this antimicrobial resistance and hence in proffering a solution.

Considering the pivotal role *E. coli* plays as a human pathogen and the numerous non-clinical reservoirs associated with it, this study therefore set out to explore the antibiotic resistance and ESBL production of *E. coli* from various sources in Aba metropolis of Abia State, Nigeria.

## Methods

### Study area and sample collection

The work was carried out within Aba metropolis in Abia State, Nigeria. Aba is a commercial city which is located at latitude of 5.1216° N and 7.3733° E longitude. Isolates were obtained from various clinical and non-clinical sources including urine, stool, wound, soil, water, abattoir effluents, animal litter and ready-to-eat food. Ethical approval was obtained from the hospital board.

### Isolation and identification of *Escherichia coli*

Clinical isolates were inoculated directly on blood agar and eosin methylene blue agar plates and incubated at 37 °C for 24 h. Non–clinical samples were serially diluted appropriately and inoculated into eosin methylene blue agar and MacConkey agar and incubated at 37 °C for 24 h. They were further sub-cultured and identified phenotypically using standard biochemical methods (Cheesbrough 2000).

### Molecular Identification of *E. coli*

Isolates were further identified using molecular methods adopted from previous studies with slight modifications (Islam et al. 2010; Rahman et al. 2017; Otokunefor et al. 2019a). In brief, the standard boiling method was employed for the isolation of the bacteria DNA. The pure isolates were boiled in 100 µL of molecular grade water for 5 min. This was followed by centrifugation at 10,000*g*/min for 5 min, and bacterial DNA which was suspended in the supernatant was separated from the sediment and was used as DNA template. *E. coli* isolates were confirmed by specific 16S rRNA gene fragments detection using the Ec16 primer pair (F 5′-GACCTCGGTTTAGTTCACAGA-3′ and R 5′-CACACGCTGACGCTGACCA-3′). The reaction mixture was prepared by adding 3 µl of the genomic DNA, 10 µl of PCR master mixtures, 1 µl of each of the primers and 5 µl of nuclease-free H_2_O to adjust the volume to 20 µl. Initial denaturation was done at 95 °C to apply the primers, which was followed by denaturation at 94 °C for 45 s. The primers were annealed at 55 °C for 45 s with extension at 72 °C for 1 min. Final extension was done at 72 °C for 5 min. Thirty cycles were completed for the total reaction. Using 2% agarose gel, the amplified PCR was resolved for 30 min by electrophoresis, and the gel was stained with ethidium bromide and visualized using a UV transilluminator.

### Antibiotic susceptibility testing

Antibiotic susceptibility testing of the identified *E. coli* was carried out by Kirby–Bauer disc diffusion method (Bauer et al. 1966). After standardization of the bacterial isolates to a turbidity level equivalent to 0.5 McFarland standard, Mueller–Hinton agar plates were inoculated with the organisms using sterile swab sticks. Following a 5-min pre-incubation, the test antibiotic multidisc was placed at the center of the petri dish. The plates were inverted and were incubated at 37 °C for 24 h. Susceptibility was then determined by comparing the zones of inhibition against a standard (CLSI 2018).

Additionally, MAR index and multidrug-resistant status of isolates were also determined from the results of the antibiotic susceptibility testing. The MAR index was calculated using the formula a/b, where "a" is the total number of antibiotics to which the organism was resistant and "b" is the total number of antibiotics against which the organisms were tested. The multidrug resistance was defined as resistance to three or more classes of antibiotics.

### Phenotypic detection of ESBL

The presence of ESBL was determined phenotypically by the double-disc synergy test. In brief, a standard suspension of the isolate was spread evenly on the surface of Mueller–Hinton agar plates. Discs of cefotaxime, ceftazidime, ceftriaxone and cefpodoxime (30 µg each) were placed at a distance of 15 mm edge to edge from a centrally placed amoxicillin–clavulanate disc containing 20 µg of amoxicillin and 10 µg of clavulanic acid. The plates were incubated at 35 °C for 24 h. The pattern of zone of inhibition was noted. Isolates that exhibited a distinct shape/size (keyhole effect) with potentiation toward amoxicillin + clavulanate disc were confirmed as ESBL producers (CLSI 2018).

### Detection of bla_TEM_, bla_SHV_ and bla_CTX-M_ genes in E. coli

The presence of three beta-lactamase gene genotypes (bla_TEM_, bla_SHV_ and bla_CTX-M_) in the *E. coli* isolates was assessed as previously described (Goudarzi et al. 2013). The primers were amplified with thermal cycling conditions for 5 min at 94 °C and 36 cycles of amplification consisting of 1 min at 94 °C, 1 min at 55 °C and 1 min at 72 °C, with 5 min at 72 °C for the final extension. Analysis of the PCR band was carried out using electrophoresis in a 1% agarose gel at 95 V for 45 min using ethidium bromide under UV irradiation.

## Results

From a total of 350 samples collected, 137 were phenotypically identified as *Escherichia coli*. Of these, only 83 isolates were confirmed to be *E. coli* using genotypic typing based on the presence of *E. coli* specific 16S rRNA fragments. Majority (52, 62.7%) of the 83 confirmed *E. coli* isolates were from non-clinical sources.

**Fig. 1:**
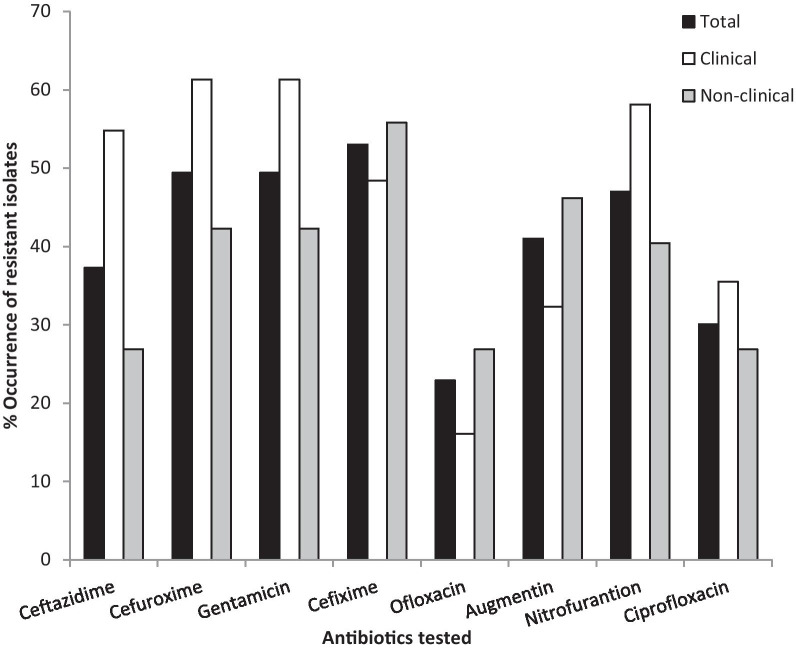
Rate of bacteria resistance to tested antibiotics

An analysis of the antibiotic susceptibility testing showed that in general, isolates exhibited mid-level resistance against the different antibiotics with resistance rates ranging from 22.9% to 53.0% (Fig. 1). For majority of the antibiotics (7/8, 87.5%), a less than 50% resistance was observed. A comparison of resistance exhibited by the different isolates based on source, however, showed clinical isolates exhibited higher levels of resistance than non-clinical isolates against majority (5/8, 62.5%) of antibiotics. Clinical isolates had resistance rates ranging from 16.1% to 61.3% with rates above 50% observed against 50% of antibiotics. The non-clinical isolates on the other hand had rates ranging from 26.9 to 55.8% with rates above 50% observed against only one antibiotic (87.5%).

An assessment of the resistance pattern of individual isolates revealed that 11 of the 83 isolates (13.3%) were fully susceptible to all antibiotics (Table 1). Surprisingly, a higher percentage of clinical isolates (16.1%) were fully susceptible as opposed to the non-clinical isolates (11.5%). A total of 51 antibiogram patterns were detected among the isolates. For the clinical isolates, 20 different patterns were identified, while 37 different patterns were identified from the non-clinical isolates. Only five of these patterns were common to the two groups of isolates.

**Table 1:** Antibiogram pattern of the isolates

S. no.	Antibiogram	Clinical	Non-clinical	No. of drug classes
1.	Pan-Susceptible	5	6	0
2.	AUG	1	5	1
3.	CPR		1	1
4.	GEN	2		1
5.	NIT	1	1	1
6.	AUG-CRX		1	2
7.	AUG-NIT		1	2
8.	CAZ-CRX	1	1	1
9.	CRX-CXM		1	2
10.	CRX-GEN	1		2
11.	CRX-OFL		1	2
12.	GEN-NIT		1	2
13.	AUG-CPR-GEN		1	3
14.	AUG-CPR-OFL		1	3
15.	AUG-CRX-CXM		1	2
16.	AUG-CRX-GEN	1		3
17.	AUG-CXM-GEN	1		3
18.	AUG-CXM-OFL		1	3
19.	AUG-GEN-NIT		2	3
20.	CAZ-CRX-NIT	1		2
21.	CAZ-CXM-GEN		2	2
22.	CPR-GEN-NIT	1		3
23.	CRX-CXM-GEN		1	2
24.	CRX-GEN-NIT	1	1	3
25.	CXM-GEN-NIT		1	3
26.	AUG-CAZ-CRX-CXM	1		2
27.	AUG-CRX-CXM-NIT		1	3
28.	CAZ-CPR-CRX-NIT	1		3
29.	CAZ-CPR-CXM-NIT		1	3
30.	CAZ-CRX-CXM-GEN		1	3
31.	CAZ-CXM-NIT-OFL		1	3
32.	CRX-CPR-CXM-OFL		1	2
33.	CRX-CXM-GEN-NIT		3	3
34.	CRX-CXM-GEN-OFL		2	3
35.	AUG-CAZ-CPR-CXM-NIT	1	1	4
36.	AUG-CAZ-CPR-CXM-OFL		1	3
37.	AUG-CAZ-CXM-NIT-OFL		1	4
38.	AUG-CPR-CRX-CXM-GEN		1	4
39.	CAZ-CRX-CXM-GEN-NIT	2		3
40.	CRZ-CPR-CXM-GEN-OFL		1	3
41.	AUG-CAZ-CPR-CRX-CXM-GEN		1	4
42.	AUG-CAZ-CPR-CRX-CXM-NIT		2	4
43.	AUG-CAZ-CRX-CXM-GEN-NIT	2		4
44.	AUG-CPR-CXM-GEN-NIT-OFL		1	5
45.	AUG-CRX-CXM-GEN-NIT-OFL		1	5
46.	CAZ-CPR-CRX-CXM-GEN-NIT	2		4
47.	CAZ-CRX-CXM-GEN-NIT-OFL		1	4
48.	AUG-CAZ-CPR-CRX-CXM-GEN-NIT	1		5
49.	AUG-CAZ-CPR-CXM-GEN-NIT-OFL		1	5
50.	CAZ-CPR-CRX-CXM-GEN-NIT-OFL	3		5
51.	AUG-CAZ-CPR-CRX-CXM-GEN-NIT-OFL	2		5

AUG, augmentin; CAZ, ceftazidime; CPR, ciprofloxacin; CRZ, cefuroxime; CXM, cefixime; GEN, gentamicin; NIT, nitrofurantoin; OFL, ofloxacin

**Fig. 2:**
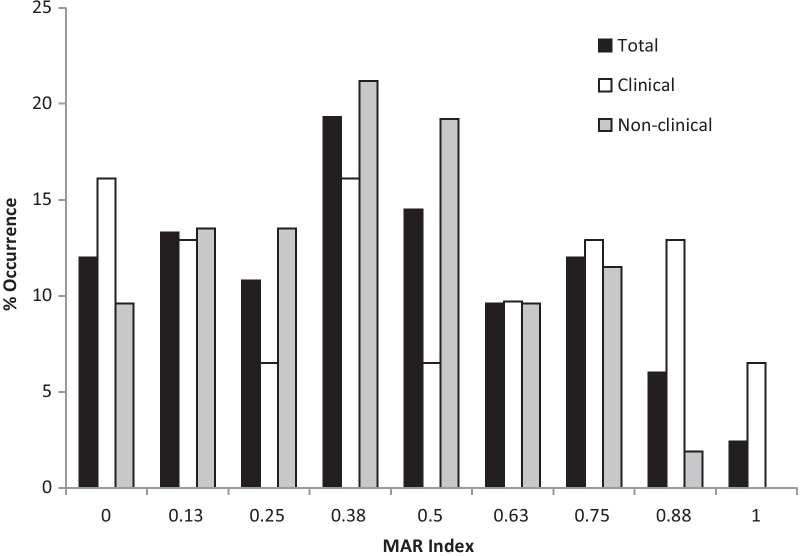
MAR index distribution occurrence of test isolates

Isolates in this study showed similar levels of distribution across the different MAR indices (Fig. 2). One difference between clinical and non-clinical isolates was in the representation among the higher MAR indices (0.75–1). A total of 20.4% of isolates were found within this range, but for the non-clinical isolates, this comprised only 13.4%, while for the clinical, it comprised 32.5% of isolates.

**Fig. 3:**
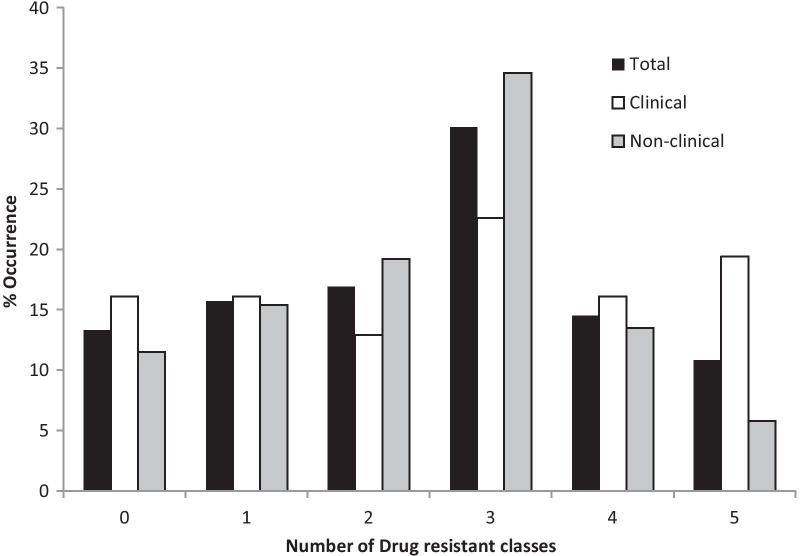
Variation in resistance levels of the different test isolates

Majority of isolates tested were multidrug resistant (55.4%), exhibiting resistance to three or more drug classes (Fig. 3). Similar levels of MDR were observed from both clinical and non-clinical isolates (58.1% and 53.9%, respectively). More of the clinical isolates, however, were resistant to all five classes of antibiotics than the non-clinical isolates (19.4% versus 5.8%).

**Fig. 4:**
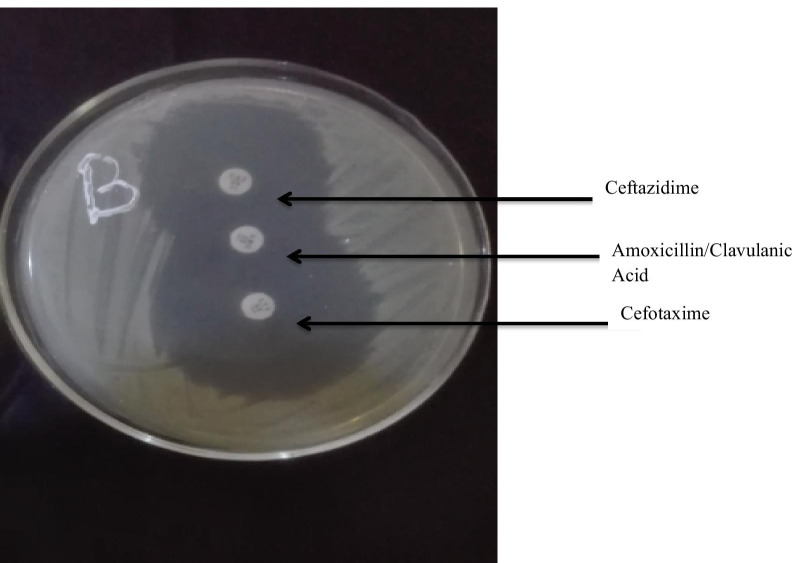
ESBL-positive isolate

**Fig. 5:**
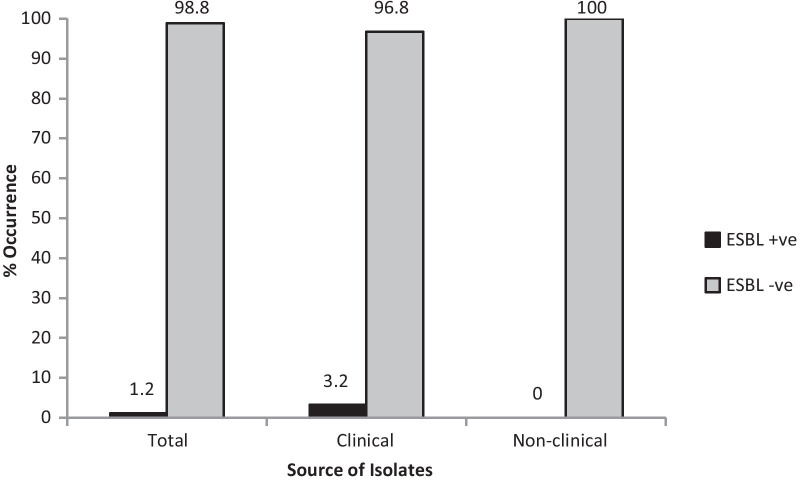
Frequency occurrence of ESBL production

Phenotypic detection of ESBL showed a low occurrence of ESBL among the test isolates. Only one isolate (1.2%) showed ESBL production as detected by the double-disc synergy test (Fig. 4). This isolate was a clinical rather than a non-clinical isolate (Fig. 5). Genotypic detection of three ESBL-related genes in all the test isolates was similar to the results of the phenotypic detection. None of the three ESBL-related genes was detected among all the test isolates.

## Discussion

*Escherichia coli* have currently been classified as one of the antibiotic-resistant organisms which are of serious clinical concern (Goudarzi et al. 2013). In addition to being one of the two predominant organisms isolated from the clinical microbiology laboratory, these isolates have been readily described in food, water and soil (Haberecht et al. 2019; Sabala et al. 2021). These could therefore serve as reservoirs which encourage the spread of resistance determinants to man. This was buttressed by results of this study reporting resistance rates of 26.9% to 55.8% among non-clinical isolates. Similar moderate rates in non-clinical *E. coli* isolates have been described by other studies (Haberecht et al. 2019; Sabala et al. 2021; Adelowo et al. 2014; Odonkor and Addo 2018). Though, in some instances, much higher rates of resistance were described either in general or to specific antibiotics (Adelowo et al. 2014; Sarker et al. 2019; Otokunefor et al. 2019b). Some of these were linked with poultry environment and thought to be a reflection of antibiotic use in these environments.

Clinical environments are generally related to higher levels of antibiotic exposure, and this is often thought to lead to the selection of resistant isolates in these environments. Quite often, resistance rates described in clinical environments are relatively high, with rates over 50% observed against a significant number of antibiotics (Kibret and Abera 2011; Igwe et al. 2016; Monira et al. 2017; Tuem et al. 2018; Pormohammad et al. 2019). This similar trend was observed in this present study.

However, unlike some previous reports from within and outside Nigeria which observed MDR rates ranging from 52 to 100% (Igwe et al. 2016; Monira et al. 2017; Makanjuola et al. 2018; Ramírez-Castillo et al. 2018; Onyeadi and Agbagwa 2019), the rate of MDR observed in this study was much lower. It was, however, similar to a 2019 study (Pormohammad et al. 2019) reporting MDR prevalence rates of 22% and 31.3% in *E. coli* from human and animal sources, respectively.

The low prevalence of ESBL production detected in this study is quite dissimilar to majority of current reports. As ESBL production has been noted to be on the rise worldwide (Lob et al. 2016; Alqasim et al. 2018), recent studies from around the globe report rates ranging from 33 to 91% (Monira et al. 2017; Alqasim et al. 2018; Hassuna et al. 2020; Pandit et al. 2020; Tufa et al. 2020). This could be a reflection of both an increase in awareness and detection practices and a genuine increase in the development of such strains. In Nigeria, rates as low as 24% and as high as 100% have been reported (Igwe et al. 2016; Agbagwa and Aminofifori 2017; Nwafia et al. 2019; Onanuga et al. 2019; Ugwu et al. 2020). A 2020 review on ESBL in Nigeria reported rates ranging from 7.5% to 82.3% with rates as low as 8.1% observed from the same region as this present study (Tanko et al. 2020). Low rates of prevalence have been linked with several regions worldwide, especially the north/eastern European countries. This low prevalence has been thought to be possibly affected by variations in specific ESBL detection methodologies, in addition to low antibiotic use (Sepp et al. 2019).

Quite often, lower ESBL rates have been observed using molecular techniques, as opposed to the phenotypic techniques. A 2019 study reported the detection of ESBL genes in only 94.7% of phenotypically identified ESBL isolates (Sepp et al. 2019). While three main ESBL genes exist, numerous genes have been linked with ESBL production. The inability therefore to detect any of the three main ESBL genes in this study points to the presence of another ESBL gene in the test isolates in this study.

## Conclusions

This study reports high levels of antibiotic resistance in both clinical and non-clinical *E. coli* isolates. Though higher rates of resistance were found among the non-clinical isolates, both group of organisms had similar levels of MDR. Strikingly, however, was the low level of ESBL producers detected in this study and the absence of the three main genotypes associated with ESBL production in this study.

## Abbreviations

ESBL: Extended-spectrum beta-lactamase
RNA: Ribonucleic acid
rRNA: Ribosomal RNA
MDR: Multidrug resistance
DNA: Deoxyribonucleic acid
PCR: Polymerase chain reaction
UV: Ultraviolet
MAR: Multiple antibiotic resistance
